# Mental health changes during and after the COVID-19 pandemic in children and adolescents with mental disorders

**DOI:** 10.1007/s00787-025-02700-1

**Published:** 2025-04-05

**Authors:** Josjan Zijlmans, Lotte van Rijn, Hekmat Alrouh, Emma Broek, Michiel Luijten, Jacintha Tieskens, Malindi van der Mheen, Hedy van Oers, Wiepke Cahn, Arnt Schellekens, DREAMS Consortium, J.K. Bird, J.K. Buitelaar, Y. de Vries, T.J. Dekkers, Y. Haveman, I. Hein, P.J. Hoekstra, H. Klip, R.J.L. Lindauer, M. Luman, M.H. Nauta, L.M.C. Jansen, L. Nijland, S. Pieters, A. Popma, W. Staal, D. van Doelen, R.R.J.M. Vermeiren, I. Visser, M. Wildschut, Tinca J. C. Polderman, Janneke R. Zinkstok

**Affiliations:** 1 Department of Child and Adolescent Psychiatry and Psychosocial Care, Amsterdam University Medical Center, Location Vrije Universiteit Amsterdam, Amsterdam, The Netherlands; 2 Amsterdam Public Health, Amsterdam University Medical Center, Mental Health, Amsterdam, The Netherlands; 3 Levvel, Academic Center for Child and Adolescent Psychiatry, Amsterdam, The Netherlands; 4 Department of Psychiatry, Donders Institute for Brain, Cognition and Behavior, Radboud University Medical Center, Nijmegen, The Netherlands; 5 Department of Psychiatry, University Medical Center Utrecht, Utrecht, The Netherlands; 6 Department of Biological Psychology, Vrije Universiteit Amsterdam, Amsterdam, The Netherlands; 7 Leiden University Medical Center Curium, Leiden, The Netherlands; 8 Child and Adolescent Psychiatry and Psychosocial Care, Amsterdam University Medical Center, Location University of Amsterdam, Emma Children’s Hospital, Amsterdam, The Netherlands; 9 Epidemiology and Data Science, Amsterdam University Medical Center, Vrije Universiteit Amsterdam, Amsterdam, The Netherlands; 10 Department of Child and Adolescent Psychiatry, Amsterdam University Medical Center, Location University of Amsterdam, Amsterdam, The Netherlands; 11 Department of Psychiatry, Brain Center, University Medical Center Utrecht, Utrecht, The Netherlands; 12 Altrecht, Institute for Mental Health Care, Utrecht, The Netherlands; 13 Nijmegen Institute for Scientist Practitioners in Addiction (NISPA), Nijmegen, The Netherlands; 14 Karakter Child and Adolescent Psychiatry University Centre, Nijmegen, The Netherlands; 15 Accare Child Study Center, Groningen, The Netherlands

**Keywords:** COVID-19, Corona virus, Pandemic, Child and adolescent psychiatry, Mental disorders, Mental health

## Abstract

The COVID-19 pandemic negatively affected child and adolescent mental health, but it is unclear which subgroups were affected most. We investigated to what extent severity and type of mental health problems during and after the pandemic were related to preexisting mental disorders in children in care at child and adolescent mental health services. We employed a repeated cross-sectional design involving data collection at seven time points (April 2020 to April 2023) in a total sample of 2,545 children (age 8–18 years). We grouped diagnostic classifications in four categories: Autism, ADHD, Anxious/Depressive disorders, and ‘Other’. Mental health was assessed with parent‐reported data on internalizing and externalizing problems and with self‐reported data from the standardized PROMIS questionnaires ‘Anxiety’, ‘Depressive symptoms’, ‘Sleep‐related impairments’, ‘Anger’, ‘Global health’, and ‘Peer relations’. We tested for main effects between diagnostic categories and for different trajectories over time. We found that mental health outcomes varied substantially between diagnostic categories, with internalizing problems being largest in children with Anxious/Depressive disorders, and externalizing problems being largest in children with Autism and ADHD. However, we found no evidence for differences between diagnostic categories in trajectories in mental health outcomes during and after the COVID-19 pandemic. The results show that during the pandemic mental health outcomes worsened over time in children and adolescents in care, and that this negative effect on mental health did not differ between children with different diagnostic classifications. Regular high-quality monitoring is vital to recognize changing trajectories of youth mental health and to adapt to crisis situations.

## Introduction

The COVID-19 pandemic has had a major impact on people’s lives, and concerns about its burden on mental health are substantial. There is converging evidence that effects on mental health problems in adults have been limited [1], but this is not the case for children and adolescents (hereafter referred to as children). Meta-analyses in general population samples showed a surge of mental health problems during the pandemic (in comparison to pre-pandemic data), with a particular increase in anxious and depressive symptoms [2, 3]. Although few studies have been performed in clinical child populations, surveys in children with various diagnoses (e.g., autism, attention deficit hyperactivity disorder [ADHD], eating disorders) suggest that mental health problems increased during the pandemic in these populations as well [4–9].

Thus far, the pursuit for factors that might predict which children were affected most by the pandemic has not been fruitful. Age and sex seem unrelated to mental health changes due to the pandemic in children [10, 11], although some studies found a slightly larger impact on adolescents compared to younger children [12, 13]. It has been suggested that some children with mental health problems may be especially susceptible to mental health effects due to the pandemic, whereas others may show surprising resilience or benefits. For example, relief of social and sensory pressure may have greater upsides for children with particular mental health problems [14]. To the best of our knowledge, no study to date has yet compared the pandemic impact on outcomes between children with different mental disorders directly.

In previous work, we examined changes in child mental health in a large clinical child sample from the start of the COVID-pandemic until approximately one year after the pandemic (lockdown) measures ended (April 2023) [9]. Our results showed that internalizing mental health problems reported by both children and parents increased throughout the pandemic and were still elevated one year after the end of the pandemic. In the current study, we investigated a large cohort of Dutch children receiving care by Child and Adolescent Mental Health Services (CAMHS). We tested if 1) self-reported and parent-reported mental health outcomes varied across mental disorders, and if 2) changes in self-reported and parent-reported mental health outcomes across time varied across mental disorders.

## Methods

### Procedure

**Fig. 1:**
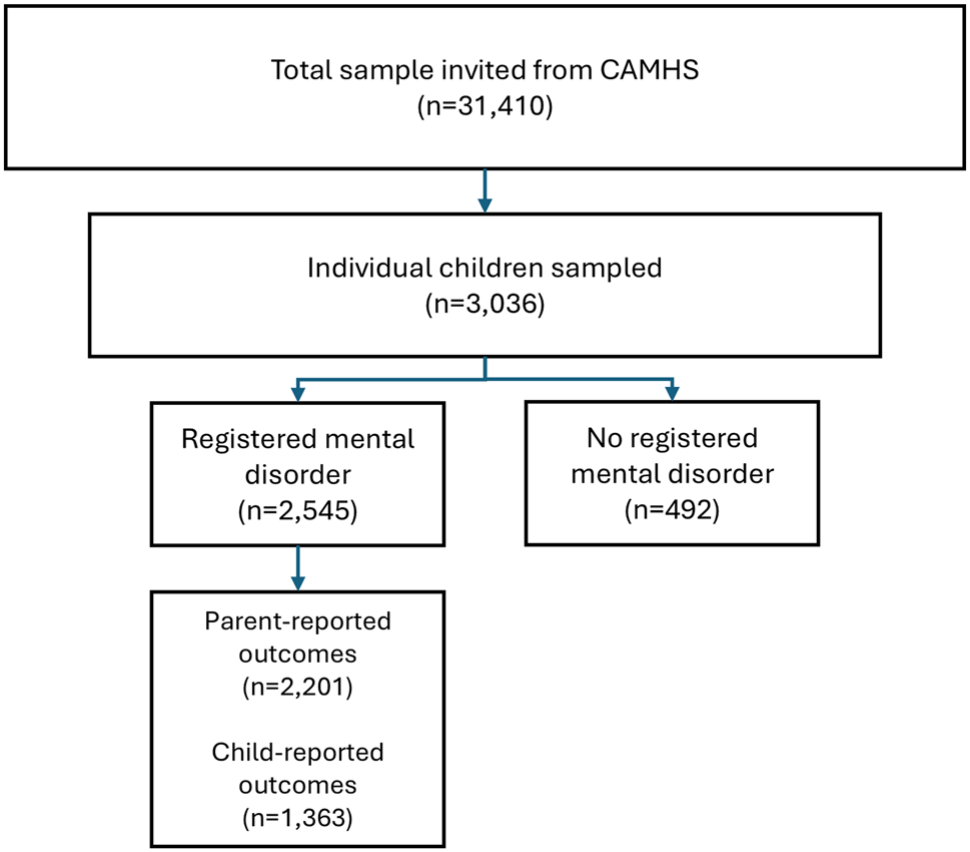
STROBE flowchart of participants

We collected data at seven time points, approximately once every six months between April 2020 and April 2023 in a repeated cross-sectional design. Parents of all children between the ages of 8 and 18 receiving CAMHS care in one of the four DREAMS centers were invited via email to participate in the study together with their child. See Fig. 1 for a flowchart of the sampling.

**Fig. 2:**
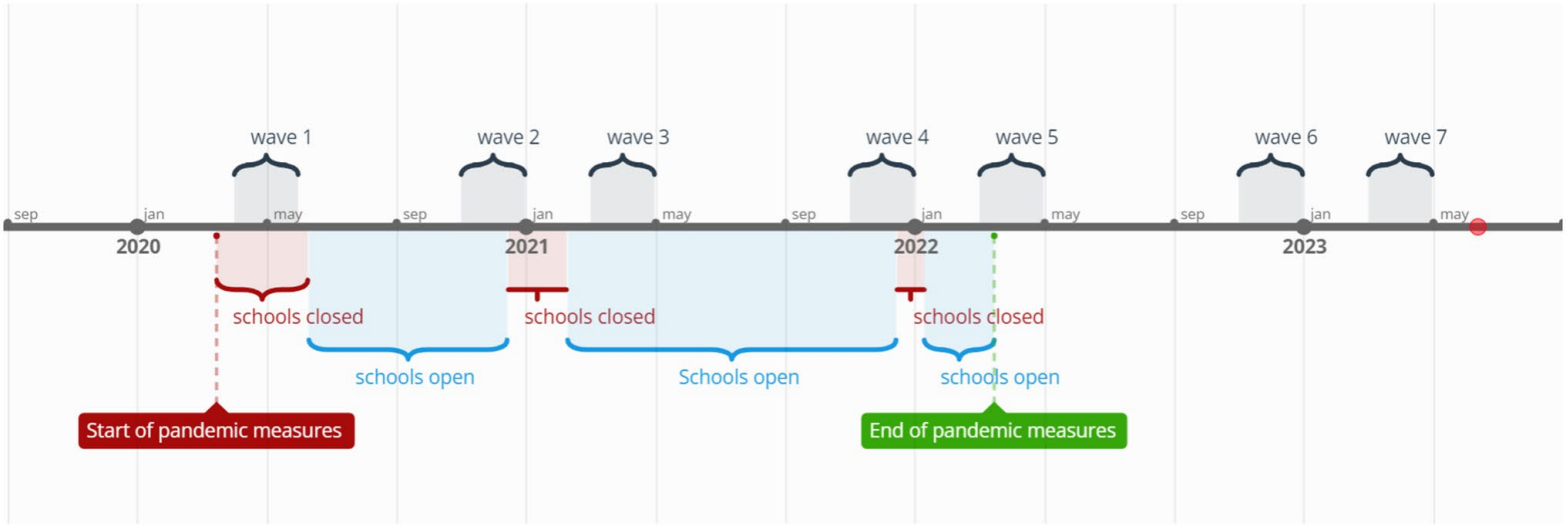
Timeline of data collection waves and pandemic measures in the Netherlands

All study data were gathered via online questionnaires via a dedicated research website. Response rates varied between 9 and 11%. During COVID-19, in the Netherlands several restrictions were in place including lockdowns, school closures, and physical distancing. Figure 2 provides an overview of the seven data waves in relation to Dutch COVID-19 restrictions at the time.

### Participants

Participants were 2,545 children aged 8 to 18 years old (mean age 13.3, SD 2.93) who received CAMHS care in one of the four Youth Mental Health Services that offer specialized care and collaborate in DREAMS (www.dreams-study.nl). See Table 1 for an overview of participant characteristics.

**Table 1:** Characteristics of the Sample

Group		Early pandemic	Late pandemic	Post pandemic
Autism	N	628	294	200
	Male	62.3%	56.8%	56.5%
	M age in years (SD)	13.09 (2.85)	13.19 (2.83)	13.25 (2.77)
	Country of birth parents (both Dutch)	91.0%	85.7%	89.5%
	Educational level parents low	3.7%	4.2%	5.0%
	Educational level parents intermediate	42.6%	41.8%	37.0%
	Educational level parents high	53.8%	54.0%	58.0%
ADHD	N	354	137	96
	Male	72.6%	69.3%	63.5%
	M age in years (SD)	12.3 (2.81)	11.60 (2.53)	12.08 (2.79)
	Country of birth parents (both Dutch)	87.7%	83.9%	90.6%
	Educational level parents low	6.3%	7.4%	6.3%
	Educational level parents intermediate	46.0%	51.5%	42.1%
	Educational level parents high	47.7%	41.2%	51.6%
Anxiety/Mood	N	251	111	88
	Male	33.1%	34.2%	37.5%
	M age in years (SD)	14.70 (2.79)	14.41 (2.74)	14.35 (2.50)
	Country of birth parents (both Dutch)	82.6%	79.2%	79.6%
	Educational level parents low	6.9%	8.9%	5.7%
	Educational level parents intermediate	41.5%	38.6%	37.5%
	Educational level parents high	51.6%	52.5%	56.8%
Other	N	220	94	72
	Male	37.7%	31.9%	37.5%
	M age in years (SD)	14.19 (2.92)	14.10 (2.78)	14.94 (2.56)
	Country of birth parents (both Dutch)	79.3%	78.2%	85.9%
	Educational level parents low	5.1%	3.4%	5.6%
	Educational level parents intermediate	38.9%	40.2%	22.5%
	Educational level parents high	56.1%	56.3%	71.8%

*N*  number of participants, *M*  mean, *SD*  standard deviation. Early pandemic = waves 1–3; April 2020-April 2021, Late pandemic = waves 4–5; November 2021-April 2022, Post pandemic = waves 6–7; November 2022-April 2023

### Measures

#### Diagnostic information

We collected primary DSM-5 diagnoses [15] as assessed in regular clinical care from the medical records of the participants. See Van der Mheen et al., 2024 for details on diagnostic procedures. We grouped the diagnoses into four categories: ADHD, Autism, Anxious/Depressive disorders, and Other (e.g., eating disorders, disruptive behavior disorders). For 16.2% of children no mental disorder was registered. We excluded these participants from the analyses.

### Socio-demographic information

We collected socio-demographic information, including age and sex of the child, and the parents’ country of birth and educational level. We defined parents’ country of birth as both parents being born in the Netherlands (yes/no). We categorized parental educational level based on the highest education level among both parents and coded this as low (primary education, lower vocational education, or lower and middle general secondary education), intermediate (middle vocational education, higher secondary education, or pre-university education), or high (higher vocational education or university).

### Parent-reported outcomes

Parents filled out the Brief Problem Monitor (BPM) from the Achenbach System of Empirical Based Assessment (ASEBA-BPM), which is a shortened version of the Child Behavior Checklist (CBCL/6–18 years) [16] that assesses behavioral and emotional problems in children as reported by their parents. Items were rated on a three-point Likert-scale, where parents rated if a statement applies to their child in the past seven days (0 = ‘not true’, 1 = ‘somewhat true’, 2 = ‘very true’). We used the internalizing and externalizing scales of the BPM. The externalizing score typically consists of seven items, but we excluded one item related to behavior at school due to data collection occurring during periods with COVID-19 restrictions when children did not attend school. The six remaining items were weighted to maintain the same range as the normal scoring system, allowing for comparison to other studies. The internalizing score was calculated using the regular six items. In line with the BPM manual, we coded missing items on the BPM as zero. If more than 20% of items were missing for a participant, they were excluded from the BPM analysis.

### Child-reported outcomes

Children filled out six measures from the Patient-Reported Outcomes Measurement Information System (PROMIS^®^) to assess self-reported aspects of health over the past seven days: Anxiety v2.0 [17], Depressive Symptoms v2.0 [17], Anger v2.0 [18], Sleep-related impairment v1.0 (including self-reported perceptions of alertness, sleepiness, and tiredness as well as perceived functional impairments during wakefulness associated with sleep problems) [19], Global health v1.0 [20], and Peer Relationships v2.0 [21].

Anger (9 items, e.g., “I was so angry I felt like yelling at somebody”) and Global health (7 items, e.g., “In general, would you say your quality of life is”) were administered as short forms. Anxiety (15 items in item bank, e.g., “I felt like something awful might happen”), Depressive Symptoms (14 items in item bank, e.g., “I could not stop feeling sad”), Sleep-related impairment (13 items in item bank, e.g., “I had problems during the day because of poor sleep”), and Peer Relationships (15 items in item bank, e.g., “My friends and I helped each other out”) were administered as Computerized Adaptive Tests (CAT), where items are selected based on responses to previously completed items, resulting in reliable scores with fewer items [22]. Most items were scored on a five-point Likert scale ranging from ‘never’ to ‘(almost) always’. Total scores were calculated by transforming item scores into T-scores ranging from 0 to 100, with a mean of 50 and a standard deviation of 10 in the original calibration sample [17]. The U.S. item parameters were used in the CAT algorithm and T-score calculations, as by PROMIS convention. The PROMIS pediatric item banks and scales have previously been validated in the Dutch population [23–27].

### Data analysis

We performed statistical analyses in IBM SPSS Statistics 28. For some participants data were available at multiple time points, but this was the case for too few participants to include these data into meaningful analyses. Therefore, we randomly selected one time point per participant to avoid dependencies in the data, and thus employed a repeated cross-sectional design. We grouped the data of seven measurements into three pandemic periods: early pandemic (wave 1–3; April 2020, November 2020, and April 2021), late pandemic (wave 4–5; November 2021 and April 2022), post pandemic (wave 6–7; November 2022 and April 2023) to increase power as subgroup sizes per diagnostic category were limited for the individual measurements. For each outcome variable, we performed an analysis of covariance (ANCOVA) to test for main effects of mental disorder and interaction effects between mental disorder and pandemic period. In all analyses, we included age and sex of the child, parental country of birth, and parental education as covariates. We report all outcomes as estimated marginal means (EMMs) of Z-scores standardized to pre-pandemic norms from the general population, assessed in independent studies (for details, see [9] We performed Bayesian analyses using JASP 0.19 [28] to assess whether null results were indicative of evidence in favor of null hypotheses or indicative of a lack of evidence in either direction. Bayes factors represent how likely the data are under the null hypothesis compared to the alternative hypothesis. For example, if BF_10_ = 5, the data are five times more likely under the alternative hypothesis than under the null hypothesis, whereas if BF_10_ = 0.2, the data are five times more likely under the null hypothesis than under the alternative hypothesis [29].

## Results

### Parent-reported outcomes (BPM)

Internalizing problems differed between diagnostic categories (*p* < 0.001), with highest scores for Anxious/Depressive, followed by Autism and Other, and lowest for ADHD. Externalizing problems differed between diagnostic categories (*p* < 0.05), with highest scores for Autism and ADHD, followed by Anxious/Depressive and Other. Internalizing problems differed between pandemic periods (*p* < 0.01), but externalizing problems did not. Interaction effects between mental disorder and pandemic period were not significant for both outcomes. Bayesian analyses indicate substantial evidence in favor of the null hypotheses with Bayes factors (model with interaction term compared to model without interaction term) *BF*_10_ = 0.026 for internalizing problems and *BF*_10_ = 0.002 for externalizing problems.

### Child-reported outcomes (PROMIS)

Anxiety (*p* < 0.001), Depression (*p* < 0.001), Sleep-related problems (*p* < 0.001), and Global Health (*p* < 0.001) differed between diagnostic categories with highest scores for Anxious/Depressive, followed by Autism, Other, and lowest scores for ADHD. Anger also differed between diagnostic categories (*p* < 0.05) with highest scores for Autism, followed by Anxious/Depressive, Other, and ADHD. Peer relations differed between diagnostic categories (*p* < 0.001), with lowest scores for Autism, followed by Other, Anxious/Depressive, and ADHD. Anxiety (*p* < 0.01), Depression (*p* < 0.05), and Global Health (*p* < 0.05) differed between pandemic periods, but Sleep-related problems, Anger, and Peer relations did not. For all outcomes, interaction effects between mental disorder and time were not significant. Bayesian analyses indicate strong evidence in favor of the null hypotheses with Bayes factors (model with interaction term compared to model without interaction term) *BF*_10_ = 0.005 for Anxiety, *BF*_10_ = 0.012 for Depression, *BF*_10_ = 0.022 for Sleep-related problems, *BF*_10_ = 0.062 for Anger, *BF*_10_ = 0.017 for Global Health, and *BF*_10_ = 0.004 for Peer relations.

**Fig. 3:**
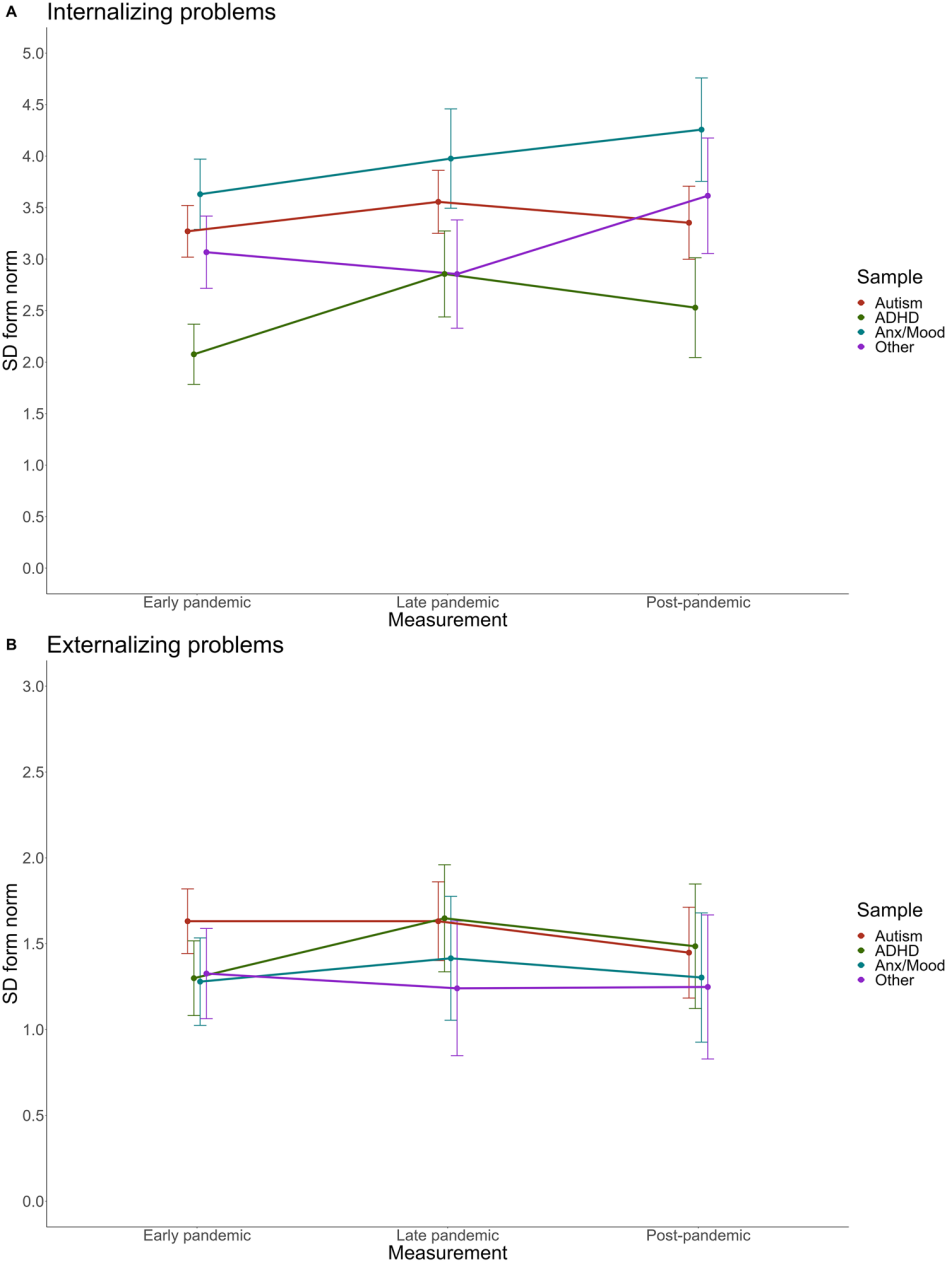
Brief Problem Monitor (BPM) estimated marginal means (EMMs) over time for different groups, represented as standard deviations from pre-pandemic norm scores

**Fig. 4:**
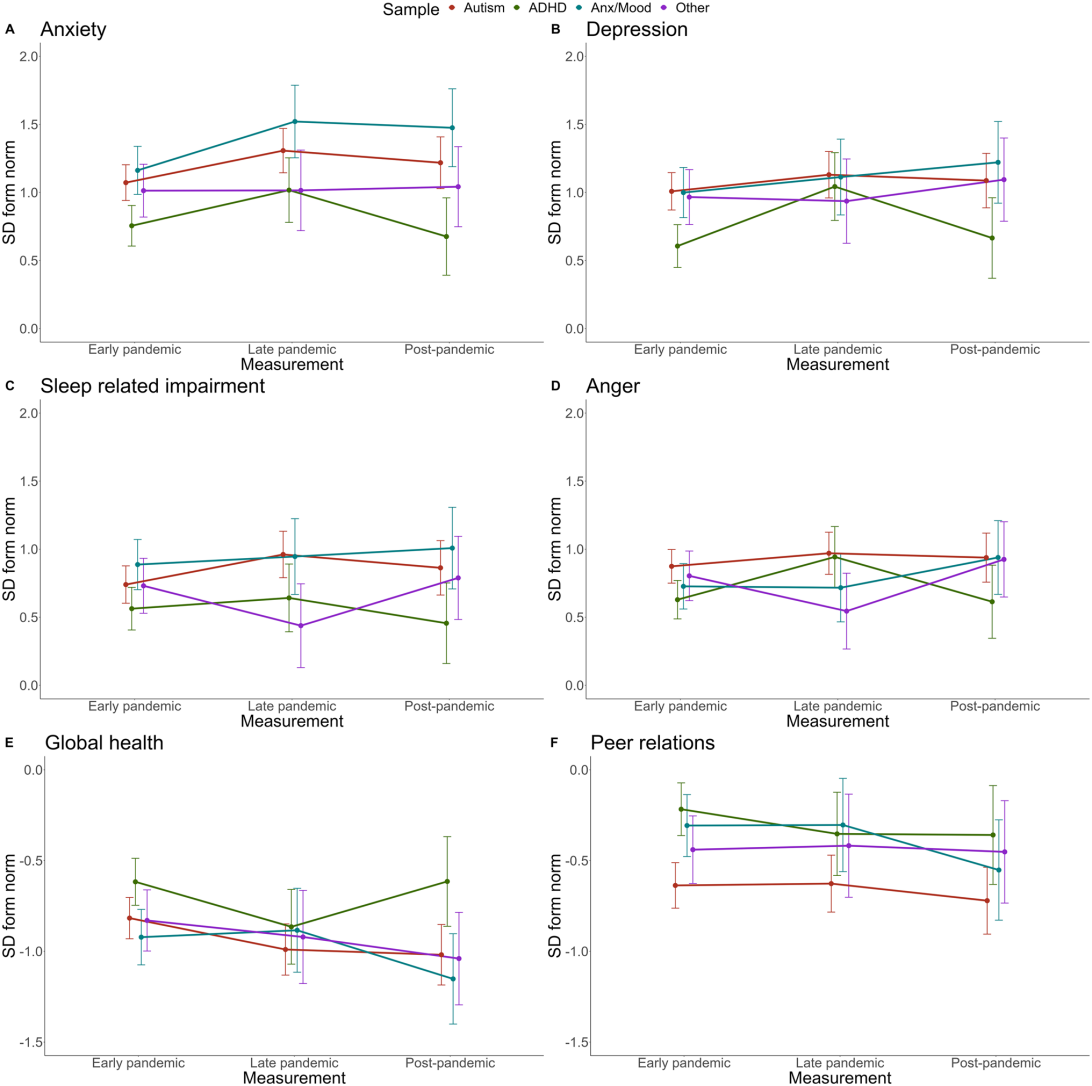
PROMIS estimated marginal means (EMMs) over time for different groups, represented as standard deviations from pre-pandemic norm scores

Figures 3 and 4 illustrate the EMMs of the different groups over time, represented as standard deviations from pre-pandemic general population norm scores. The exact statistics are reported in Table 2.

**Table 2:** BPM and PROMIS standardized estimated marginal means (EMM) and standard errors

	Cohort		1 Early pandemic	2 Late pandemic	3 Post pandemic
Parent-reported outcomes	Autism	BPM Internalizing	3.27 (0.13)	3.56 (0.16)	3.35 (0.18)
		BPM Externalizing	1.63 (0.10)	1.63 (0.12)	1.45 (0.14)
	ADHD	BPM Internalizing	2.08 (0.15)	2.86 (0.21)	2.53 (0.25)
		BPM Externalizing	1.30 (0.11)	1.65 (0.16)	1.49 (0.19)
	Anxiety/Mood	BPM Internalizing	3.63 (0.17)	3.98 (0.25)	4.26 (0.26)
		BPM Externalizing	1.28 (0.13)	1.42 (0.18)	1.30 (0.19)
	Other	BPM Internalizing	3.07 (0.18)	2.86 (0.27)	3.62 (0.29)
		BPM Externalizing	1.33 (0.13)	1.24 (0.20)	1.25 (0.21)
Child-reported outcomes	Autism	Anxiety	1.07 (0.07)	1.31 (0.08)	1.22 (0.10)
		Depressive symptoms	1.01 (0.07)	1.13 (0.09)	1.09 (0.10)
		Sleep-related impairments	0.74 (0.07)	0.96 (0.09)	0.86 (0.10)
		Anger	0.87 (0.06)	0.97 (0.08)	0.94 (0.09)
		Global health	−0.82 (0.06)	−0.99 (0.07)	−1.02 (0.09)
		Peer relations	−0.64 (0.06)	−0.63 (0.08)	−0.72 (0.09)
	ADHD	Anxiety	0.76 (0.08)	1.02 (0.12)	0.68 (0.15)
		Depressive symptoms	0.61 (0.08)	1.04 (0.13)	0.67 (0.15)
		Sleep-related impairments	0.56 (0.08)	0.64 (0.13)	0.46 (0.15)
		Anger	0.63 (0.07)	0.94 (0.11)	0.61 (0.14)
		Global health	−0.62 (0.07)	−0.87 (0.11)	−0.62 (0.13)
		Peer relations	−0.22 (0.07)	−0.35 (0.12)	−0.36 (0.14)
	Anxiety/Mood	Anxiety	1.16 (0.09)	1.52 (0.14)	1.48 (0.15)
		Depressive symptoms	1.00 (0.09)	1.11 (0.14)	1.22 (0.15)
		Sleep-related impairments	0.89 (0.09)	0.95 (0.14)	1.01 (0.15)
		Anger	0.73 (0.09)	0.72 (0.13)	0.94 (0.14)
		Global health	−0.92 (0.08)	−0.88 (0.12)	−1.15 (0.13)
		Peer relations	−0.31 (0.09)	−0.30 (0.13)	−0.55 (0.14)
	Other	Anxiety	1.01 (0.10)	1.02 (0.15)	1.04 (0.15)
		Depressive symptoms	0.97 (0.10)	0.94 (0.16)	1.09 (0.16)
		Sleep-related impairments	0.73 (0.10)	0.44 (0.16)	0.79 (0.16)
		Anger	0.80 (0.09)	0.55 (0.14)	0.93 (0.14)
		Global health	−0.83 (0.09)	−0.92 (0.13)	−1.04 (0.13)
		Peer relations	−0.44 (0.10)	−0.42 (0.15)	−0.45 (0.14)

Scores represent the constructs. As such, higher scores indicate more symptoms. For Global health and Peer relations, higher scores indicate better functioning

## Discussion

In previous work, we showed increases in mental health problems over the course of the COVID-19 pandemic (April 2020 – April 2023) in children in psychiatric care [9]. In the current study, we assessed within the same sample to what extent severity of mental health problems differed between diagnostic categories, and whether these outcomes were differently affected by the COVID pandemic across these diagnostic categories. As expected, we found that overall problems varied substantially between diagnostic categories with internalizing problems most prevalent in Anxiety/Depressive disorders, and externalizing problems most prevalent in ADHD and Autism. In other words, the type of increased mental health problems aligned overall with mental disorders as diagnosed in clinical practice. Crucially, we found no evidence for different trajectories of diagnostic groups over time, suggesting that on average children with different psychiatric classifications were equally negatively impacted during the COVID-19 pandemic.

The lack of differences in trajectories for different diagnostic categories mirrors the lack of evidence for differences in trajectories for other moderators such as age, sex, or educational attainment [9, 11]. These findings suggest that during the pandemic mental health of children decreased in general, rather than in specific subgroups. Of course, the pandemic has impacted people in different ways, and it may be the case that putative differences exist on a more individual level, or depend on broader factors such as family, neighborhood, or school. For example, early in the pandemic, economic concerns of the parents were found to be related to differences in pandemic impact on child mental health [30]. Likewise, parental stress and mental health may mediate or moderate pandemic effects on child mental health [31]. Alternatively, the impact of the pandemic may have been substantial in specific subgroups, but the coping response by caregivers, clinicians, and children themselves may have been effective in mitigating the negative impact.

Another important factor that influences the interpretation of our findings concerns the changes in mental health care itself due to the pandemic. A substantial portion of mental health care transitioned to telehealth during the pandemic, which impacted intakes, procedures, and treatments [32]. In addition, the workload, stress, and mental health problems of mental health care workers increased [33]. Such factors may have affected the accessibility of care and possibly have influenced the patient population we studied. Although we controlled for background variables and the proportion of different diagnoses did not substantially change over time, we cannot rule out that this may have contributed to our results.

This study benefits from data from a large sample of children in psychiatric care in the Netherlands and relies on both child-reported and parent-reported data. The findings of parent ratings and self-reports are congruent, suggesting that different sources of information converge towards similar outcomes. As response rates were limited, there is a risk of selection bias that may have impacted results. Although the sample is demographically representative of its population [9, 34], we cannot rule out that people more impacted by the pandemic tended to participate more or less in the study. Further, not all diagnostic categories were represented in this study since sample sizes would become too low to perform meaningful analyses, hence, our findings cannot be generalized to other specific mental disorders. Likewise, our sample consists of children in specialized psychiatric care and findings may not generalize to broader child populations with mental health problems. In addition, for our analyses we used only three time points to retain sufficient power, but thereby also increased the time spans of measurements, and therefore we cannot rule out differences between and within smaller time scales. Finally, no pre-pandemic data was available and thus we cannot rule out that the acute impact of the pandemic may have been different for children with different diagnoses.

In sum, we found no differences between diagnostic categories in mental health changes over time from the start to a year after the COVID-19 pandemic. Our data suggest that having a specific mental disorder did not pose a risk factor for worse outcomes during and after the pandemic compared to other diagnoses. We stress the need for regular, high-quality monitoring of child mental health to better understand changes that occur and to be able to better adapt to crisis situations.

## Data Availability

Data are available upon reasonable request.
